# Repeated Latency Prolongation of the Orbicularis Oris Muscle During Intraoperative Transcranial Facial Motor Evoked Potential Monitoring: A Case Report

**DOI:** 10.7759/cureus.104803

**Published:** 2026-03-07

**Authors:** Yudai Morisaki, Yasuhiro Takeshima, Kengo Yamada, Tsunenori Takatani, Ichiro Nakagawa

**Affiliations:** 1 Department of Neurosurgery, Nara Medical University, Kashihara, JPN; 2 Department of Anesthesiology, Nara Medical University, Kashihara, JPN

**Keywords:** facial nerve function, intraoperative neuromonitoring, latency, transcranial facial motor evoked potential, vestibular schwannoma

## Abstract

Intraoperative transcranial facial motor evoked potential (Tc-fMEP) monitoring is widely used to preserve facial nerve function during vestibular schwannoma surgery. Although amplitude reduction is commonly recognized as an alarm sign, latency changes have rarely been emphasized. We describe the case of a man in his 50s with neurofibromatosis type 2 who developed progressive growth of a left vestibular schwannoma. The tumor caused cerebellar compression and gait unsteadiness, and surgical resection was performed with Tc-fMEP monitoring. During the first surgery, only a partial resection was achieved due to intraoperative cerebellar swelling. Tc-fMEP amplitudes recorded from the orbicularis oculi and orbicularis oris muscles remained stable; however, latency prolongation of the orbicularis oris response appeared near the end of the procedure. Two weeks later, the patient developed mild facial paresis associated with intratumoral hemorrhage and tumor enlargement, necessitating reoperation. During the second surgery, latency prolongation of the orbicularis oris Tc-fMEP was again observed from the early stages, reproducing the previous finding, without further intraoperative changes. Facial nerve function improved during the six-month follow-up after the second surgery. In this rare case, we demonstrate reproducible Tc-fMEP latency prolongation suggestive of impaired neural conduction during vestibular schwannoma surgery. Latency prolongation of facial nerve motor evoked potentials, together with amplitude reduction, may represent a potential indicator of facial nerve functional impairment.

## Introduction

In vestibular schwannoma (VS), transcranial facial motor evoked potential (Tc-fMEP) monitoring is widely used for intraoperative assessment of facial nerve function, and a decrease in amplitude is well known to correlate with postoperative facial nerve dysfunction [1]. While amplitude reduction in Tc-MEPs is widely accepted as a critical indicator of impending neural injury, the clinical significance of latency prolongation remains less defined. In the field of spinal cord monitoring, latency delays are often attributed to focal demyelination or selective damage to fast-conducting fibers [2,3].

Regarding cranial nerve monitoring, some studies on the vagus and accessory nerves have suggested that latency shifts may precede amplitude changes as early markers of mechanical stress [4]. However, in the context of vestibular schwannoma surgery, most literature focuses on the facial motor evoked potential (MEP) amplitudes. Few studies have specifically quantified the latency prolongation of the facial nerve, and a definitive correlation between intraoperative latency shifts and postoperative functional decline has yet to be established [5]. This report aims to bridge this gap by detailing a case where repeated latency prolongation was observed.

## Case presentation

The patient was a man in his 50s with neurofibromatosis type 2 (NF2). He had undergone surgery for C4-6 spinal neurinoma 20 years ago, with no neurological sequelae. He had undergone Gamma Knife radiosurgery for a right vestibular schwannoma 19 years ago. The untreated left vestibular schwannoma gradually enlarged, causing gait unsteadiness due to cerebellar compression, and surgical resection was performed.

Preoperative neurological findings

The patient was conscious and alert, with no facial sensory abnormalities and no facial nerve paralysis. He used hearing aids because of bilateral hearing loss. No swallowing disorder was observed.

Preoperative radiological findings

**Figure 1:**
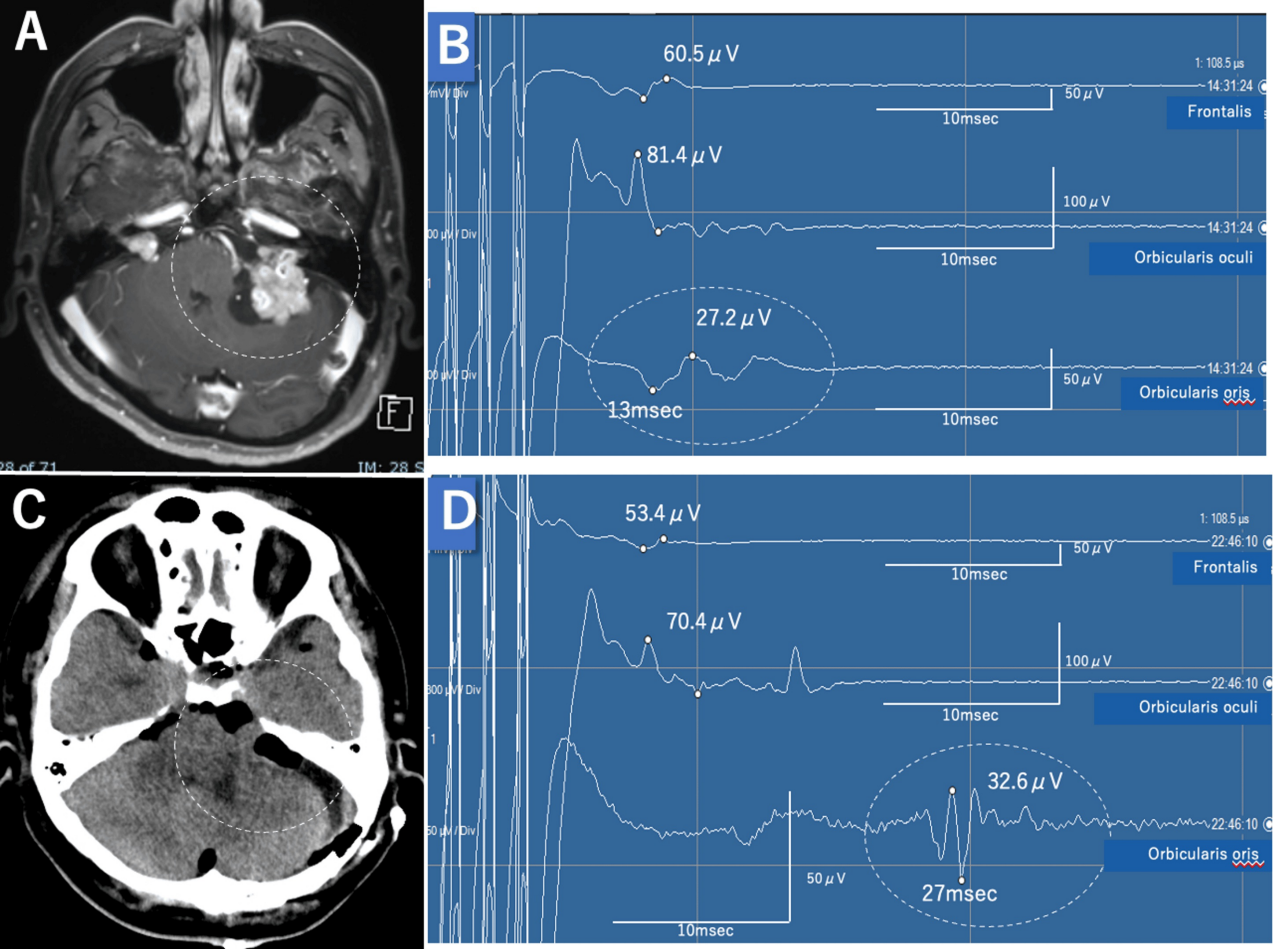
Perioperative findings of the first surgery. A: Preoperative contrast MRI shows bilateral vestibular schwannomas; the left one is 46 mm in size, with brainstem compression (dotted circle). B: Intraoperative transcranial facial motor evoked potential (Tc-fMEP) baseline waveform. The orbicularis oris muscle has a latency of 13 ms (dotted circle). C: Postoperative CT image shows partial removal of the left vestibular schwannoma (dotted circle). D: Intraoperative Tc-fMEP waveform after tumor removal. The orbicularis oris muscle has an extended latency of 27 ms (dotted circle).

Contrast-enhanced cranial MRI revealed a 46 × 34 mm tumor with multiple cysts in the left cerebellopontine angle, extending into the internal auditory canal (Figure 1, Panel A). A 7 × 10 mm tumor was also present in the right cerebellopontine angle. No hydrocephalus was observed. While these radiological features were highly suggestive of a large vestibular schwannoma, the differential diagnosis for a cystic lesion in the cerebellopontine angle includes cystic meningioma, epidermoid cyst, or schwannomas of the lower cranial nerves. In the context of NF2, the possibility of multiple or atypical presentations of cranial nerve tumors was considered; however, the tumor’s origin from the internal auditory canal and its characteristic enhancement pattern strongly supported the diagnosis of vestibular schwannoma.

Setting of intraoperative neuromonitoring

Intraoperative facial nerve monitoring included Tc-fMEPs, intermittent electrical stimulation, and free-running spontaneous electromyograms recorded from the orbicularis oculi and orbicularis oris muscles. The Tc-fMEP monitoring settings were as follows: a screw electrode served as the stimulating electrode, and anodal stimulation was applied on the affected side at C3 and C4. Recorded parameters included a low cut filter of 10-100 Hz, high-cut filter of 1-3 kHz, one addition (four-stimulus train), an interstimulus interval of 1.5-1.7 ms, a pulse width of 0.05-0.5 ms (constant voltage: 0.05 ms, constant current: 0.2-0.5 ms), an analysis time of 50-200 ms, and a sensitivity of 10-1000 μV/div. The stimulation intensity was gradually increased from 10 to 90 mA. The normal ranges and warning criteria for Tc-fMEP monitoring amplitude and latency are shown in Table 1.

**Table 1:** Reference values for intraoperative facial motor evoked potential.

Parameter	Reference range (normal)	Warning threshold (abnormal)	Description
Amplitude (μV/mV)	≥50% of baseline	<50% reduction from baseline	Significant if amplitude drops by half
Latency (ms)	Within 10% of baseline	>10% prolongation from baseline	Significant if wave is delayed by >10%

The patient was managed with total intravenous anesthesia using propofol. Target-controlled infusion (TCI) was used for propofol administration. Anesthesia was induced with propofol (target plasma concentration of 3-4 µg/mL with TCI) and remifentanil. Rocuronium (0.6-1.0 mg/kg) was administered to facilitate intubation. Anesthetic maintenance was performed with propofol (2.0-3.5 µg/mL of TCI), remifentanil (0.2-0.5 µg/kg/minute), and intermittent bolus injections of fentanyl. An intermittent bolus injection of anesthesia-related drugs was avoided during surgical maneuvers at risk of damage to the motor tract. Depth of anesthesia was adjusted to maintain the appropriate depth of anesthesia, with the bispectral index ranging from 40 to 60.

Intraoperative findings

Surgery was performed via a lateral suboccipital approach. The tumor was decompressed internally, and intermittent electrical stimulation revealed that the facial nerve ran ventrally and cranially. The posterior wall of the internal auditory canal was removed, followed by excision of the intracanalicular tumor. The tumor was prone to bleeding, making hemostasis difficult. Cerebellar swelling occurred during surgery, and only partial tumor removal was achieved. Intraoperative Tc-fMEP monitoring showed no change in the amplitudes of the orbicularis oculi and orbicularis oris muscles; however, latency prolongation of the orbicularis oris was observed (13 ms to 27 ms) toward the end of the operation (Figure 1, Panels B, C). During the first surgery, as the latency of the orbicularis oris muscle was significantly prolonged, the decision was made to stop the surgery and wait for the nerves to recover.

Postoperative course

**Figure 2:**
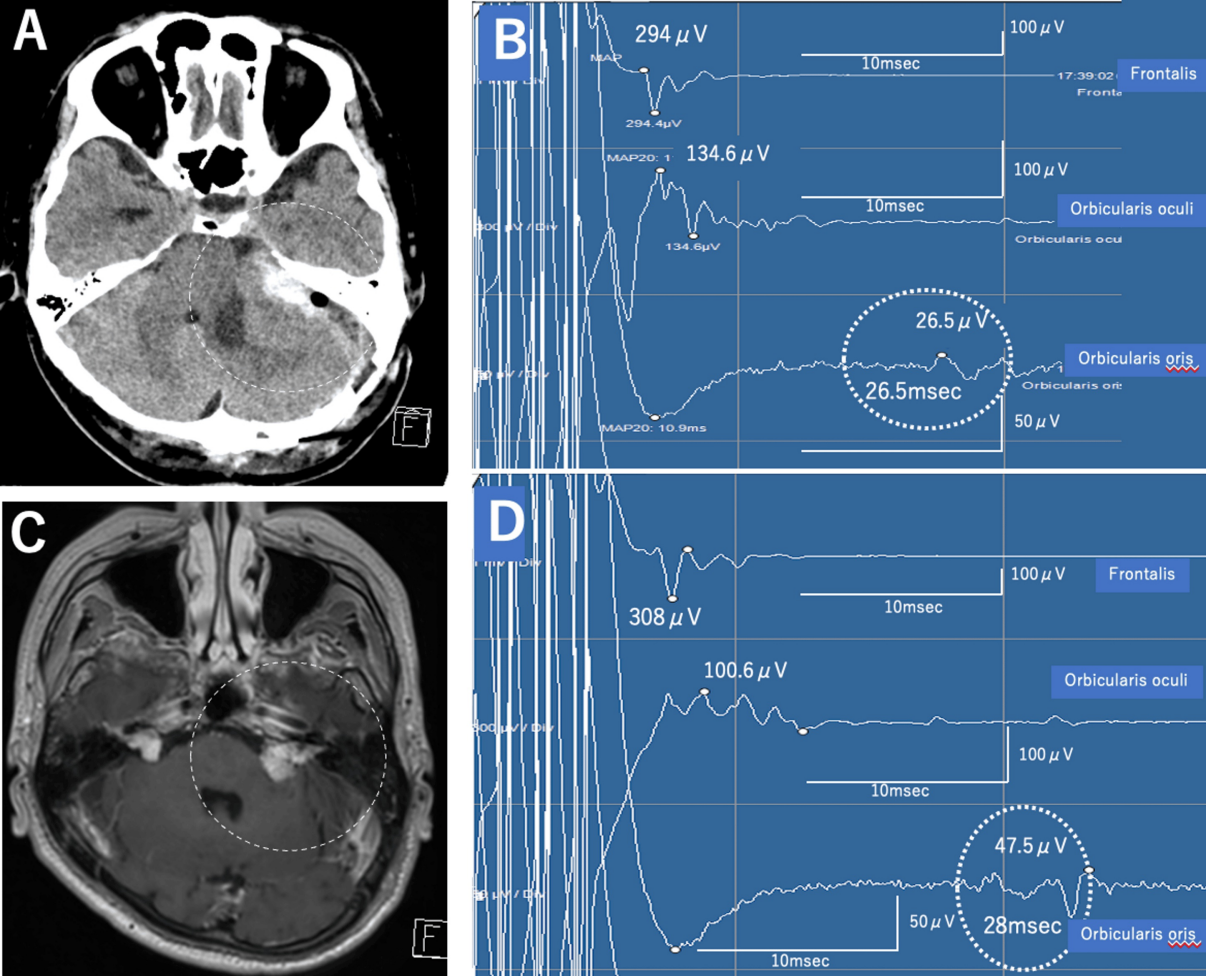
Perioperative findings of the second surgery. A: The second preoperative CT shows the tumor has regrown, and intratumoral bleeding is evident (dotted circle). B: Intraoperative transcranial facial motor evoked potential (Tc-fMEP) baseline waveform. Prolonged latency of the orbicularis oris muscle (26.5 ms) is evident. Red circle: Waveform of the orbicularis oris muscle C: Postoperative contrast MRI at six months shows that most of the tumor has been removed, and pressure on the brain stem has been relieved (dotted circle). D: Final intraoperative waveform. Prolonged latency of the orbicularis oris muscle (28 ms) is evident. Red circle: Waveform of the orbicularis oris muscle.

There was no immediate difference between the left and right sides of the face, and a head CT showed partial tumor removal (Figure 1, Panel D). Two weeks later, the patient developed mild functional decline of the left orbicularis oris muscle (House-Brackmann grade II). During this period, the residual tumor enlarged with intratumoral hemorrhage (Figure 2, Panel A). Symptoms of increased intracranial pressure due to obstructive hydrocephalus also developed, and reoperation was performed.

Reoperation findings and intraoperative neuromonitoring

The surgery was performed using the same settings as the initial surgery. Ventricular drainage was performed first, and the craniotomy was expanded laterally and caudally to facilitate cerebrospinal fluid drainage. Tumor detachment from the cerebellum and internal decompression were repeated, achieving sufficient tumor reduction. Intraoperative Tc-fMEP monitoring reproduced latency prolongation (26-28 ms) of the orbicularis oris response observed at the end of the first surgery, beginning early in the second surgery (Figure 2, Panel B). Approximately 80% of the tumor was removed, and the procedure was completed. No decrease in the amplitudes of the orbicularis oculi and orbicularis oris muscles was observed, and the latency of the orbicularis oris response remained prolonged at 28 ms at the end of tumor removal (Figure 2, Panel C).

Postoperative course

The decreased function of the orbicularis oris muscle persisted without further deterioration. Thereafter, the condition improved over time, and the patient was discharged from the hospital, able to walk independently, with a House-Brackmann grade I score. Six months after surgery, facial movement was symmetric, and contrast-enhanced MRI showed sufficient tumor reduction. Gamma Knife radiosurgery is planned for the residual tumor (Figure 2, Panel D). The intraoperative Tc-fMEP data for the first and second surgeries are shown in Table 2.

**Table 2:** Intraoperative transcranial facial motor evoked potential (Tc-fMEP) monitoring results. Maintenance rate is calculated as (final amplitude/Baseline amplitude) × 100.

Surgery	Muscle	Parameter	Baseline	Final	Maintenance rate (%)
First surgery	Frontalis	Amplitude (uV)	60.5	53.4	88.30%
		Latency (ms)	11	11	-
	Orbicularis oculi	Amplitude (uV)	81.4	70.4	86.50%
		Latency (ms)	10	11	10.00%
	Orbicularis oris	Amplitude (uV)	27.2	32.6	119.90%
		Latency (ms)	13	27	107.70%
Second surgery	Frontalis	Amplitude (uV)	294	308	104.80%
		Latency (ms)	10	10	-
	Orbicularis oculi	Amplitude (uV)	134.6	100.6	74.70%
		Latency (ms)	11	12	9.10%
	Orbicularis oris	Amplitude (uV)	26.5	47.5	179.20%
		Latency (ms)	26.5	28	5.70%

During the first surgery, the Tc-fMEP latency of the orbicularis oris muscle showed a dramatic prolongation from 13 ms (baseline) to 27 ms (end of procedure), representing a 108% increase. At the start of the second surgery, this latency remained prolonged at a baseline of 26.5 ms and reached 28 ms by the end of the procedure, indicating a persistent delay in neural conduction.

## Discussion

Preserving facial nerve function during vestibular schwannoma surgery is critical to patients’ postoperative quality of life [6]. Intraoperative Tc-fMEP monitoring is widely used to preserve facial nerve function [7,8]. Generally, in Tc-fMEP monitoring, amplitude reduction is used as a warning criterion, whereas little attention has been paid to latency. In addition, reports on the accuracy of Tc-fMEP based solely on amplitude reduction are inconsistent [1,9,10]. Therefore, reliance on amplitude changes alone has limitations for evaluating facial nerve function. By refining Tc-fMEP monitoring to improve accuracy and achieve stable waveform acquisition, we were able to detect latency prolongation. In this report, we further discuss the relationship between latency prolongation and facial nerve function.

The reported success rate of Tc-fMEP monitoring ranges from approximately 40-90%, and obtaining stable waveforms during surgery remains challenging [11]. With improved Tc-fMEP accuracy that allows stable waveform acquisition, latency prolongation may also be detected and serve as an additional indicator of reduced facial nerve function. As noted in a previous report, we applied several methods to improve Tc-fMEP accuracy [12]. First, the anode and cathode are placed at C3 and C4. Because C3 and C4 are close to the facial motor area, stimulation is easily transmitted. Furthermore, as reported by Tomio et al., the C3-C4 montage more accurately stimulates the facial motor cortex than the C3-Cz (C4-Cz) montage [13]. Second, monophasic monitoring using constant current differs from biphasic monitoring in that contralateral stimulation can serve as a control, facilitating exclusion of sliding currents. As previously reported, we believe that we were able to enable stable waveforms using various methods to reduce noise and sliding currents, thus reproducing the latency extension observed in this study [12].

In motor nerve evoked potentials, amplitude reflects the number of muscle fibers responding to nerve stimulation, whereas latency indicates the time required for the stimulus to reach the neuromuscular junction. In general, amplitude and latency of motor nerve evoked potentials are important indicators of neural function. Studies using stimulating electrodes along the facial nerve have shown that nerve conduction velocity is reduced, i.e., latency is prolonged, in patients with facial nerve paralysis [14]. In intraoperative neuromonitoring, prolonged latency during proximal facial nerve stimulation has been reported to indicate nerve-tumor adhesion or reduced nerve function [10]. During the first operation, the latency of the orbicularis oris response increased from 13 ms to 27 ms. Facial nerve fibers consist of multiple branches and motor units, and Tc-fMEPs are recorded as compound muscle action potentials. This may produce polyphasic waveforms, and partial neural injury with demyelination may manifest as latency prolongation. Experimental studies in rats have demonstrated that cochlear nerve demyelination causes latency prolongation in auditory brainstem responses [15]. In addition, nerve conduction studies in demyelinating disorders, such as Guillain-Barré syndrome, commonly show prolonged motor latencies [16]. The degree of latency prolongation observed in this case is remarkable. While a 10% increase in latency is typically considered a warning sign in other cranial nerve monitoring [4], our patient exhibited a 108% increase that did not recover between the two procedures. This suggests that such extreme latency shifts may reflect focal demyelination or structural changes in the facial nerve rather than transient surgical stress. The persistence of this delay at the second surgery’s baseline provides objective evidence of a permanent alteration in the nerve’s electrophysiological profile, even in the absence of complete functional loss. There was no immediate postoperative functional decline; however, mild dysfunction of the orbicularis oris muscle developed before the second operation, likely due to subsequent intratumoral hemorrhage. Due to tumor size and intratumoral bleeding, the clinical condition changed between the immediate postoperative period and the pre-second operation period, making the relationship between intraoperative neuromonitoring findings and facial nerve function unclear. However, the observation that latency prolongation occurred only in the orbicularis oris muscle, along with delayed functional decline confined to the same muscle, suggests a localized functional impairment. Latency measurement error is several milliseconds in visual evoked potentials [17] and less than 1 ms in MEPs [18], indicating high reproducibility. However, no studies have specifically evaluated latency measurement error in facial nerve MEPs, highlighting the need for further data accumulation.

During the second intraoperative monitoring, a waveform of the orbicularis oris muscle was observed with a latency of 26-28 ms from the start of monitoring, and as in the first surgery, an extension of the latency was observed. It is unclear whether this reproducible latency prolongation in the same patient is related to the actual functional decline; however, it suggests a nerve conduction disorder, such as demyelination. Further research is required; however, waveform latency prolongation may serve as a warning sign of facial nerve functional impairment.

We added a discussion to explain why latency prolongation was observed only in the orbicularis oris muscle. Anatomically, the facial nerve forms a single trunk from its exit at the brainstem to the internal auditory canal and contains mixed motor fibers; functional separation of fibers to individual muscles before entering the internal auditory canal has not been demonstrated [19]. Previous electrophysiological studies have reported higher monitoring accuracy in the orbicularis oris muscle than in the orbicularis oculi muscle [20], which may explain why latency prolongation was detectable only in the orbicularis oris muscle in this case. Postoperative weakness was also limited to the orbicularis oris muscle; however, a causal relationship with intraoperative monitoring changes cannot be established without further case accumulation. Facial nerve fibers generate compound action potentials with polyphasic waveforms, and partial neural injury with demyelination may result in latency prolongation. In this case, monitoring changes occurred during surgical manipulation remote from the facial nerve, making thermal injury unlikely. Stretching or traction of the facial nerve during tumor manipulation may have contributed to the observed latency prolongation. Regarding NF2, although the patient had multiple schwannomas, tumors in remote locations, such as the spinal cord, were unlikely to have directly affected the facial nerve MEPs. Intraoperatively, tumors were also identified deep within the internal auditory canal, suggesting possible coexistence of vestibular and cochlear schwannomas. No baseline latency prolongation was observed during the first surgery; however, anatomical alteration of the facial nerve in the setting of multiple schwannomas may have increased vulnerability to traction, potentially contributing to the observed latency prolongation.

This report has limitations. It describes a single case, and a direct causal relationship between latency prolongation and facial nerve dysfunction cannot be established. The postoperative facial palsy after the first surgery was most likely attributable to intratumoral hemorrhage rather than direct intraoperative nerve injury. Further studies with larger cohorts are required to validate whether such extreme latency shifts can be consistently linked to specific histological types of nerve injury.

## Conclusions

We reproducibly observed prolonged latency of Tc-fMEPs in the orbicularis oris muscle during two surgeries in the same patient. With accurate intraoperative monitoring, latency prolongation of facial nerve MEPs, together with amplitude reduction, may serve as a potential indicator of facial nerve functional impairment.
